# Visualization of chikungunya virus infection *in vitro* and *in vivo*

**DOI:** 10.1080/22221751.2019.1682948

**Published:** 2019-11-04

**Authors:** Hong-Lei Zhang, Hao-Long Dong, Ya-Nan Zhang, Lin-Lin Xu, Cheng-Lin Deng, Xiao-Feng Li, Xiao-Dan Li, Han-Qing Ye, Zhi-Ming Yuan, Cheng-Feng Qin, Bo Zhang

**Affiliations:** a Key Laboratory of Special Pathogens and Biosafety, Wuhan Institute of Virology, Center for Biosafety Mega-Science, Chinese Academy of Sciences, Wuhan, People’s Republic of China; b College of Animal Science and Veterinary Medicine, Henan Agricultural University, Zhengzhou, People’s Republic of China; c State Key Laboratory of Pathogen and Biosecurity, Beijing Institute of Microbiology and Epidemiology, Academy of Military Medical Sciences, Beijing, People’s Republic of China; d Guangzhou Eighth People’s Hospital, Guangzhou Medical University, Guangzhou, People’s Republic of China; e School of Medicine, Hunan Normal University, Changsha, People’s Republic of China; f Drug Discovery Center for Infectious Disease, Nankai University, Tianjin, People’s Republic of China

**Keywords:** Chikungunya virus, iRFP, reporter virus, live imaging, mice

## Abstract

Chikungunya virus (CHIKV), a mosquito-borne alphavirus, has become an important re-emerging pathogen with its rapid spread to many non-endemic areas. The lack of effective vaccines and antiviral agents is largely attributed to the elusive infection and dissemination dynamics *in vivo*. In this study, we designed and developed a novel, replication-competent, CHIKV reporter virus (CHIKV-iRFP) encoding a near infrared fluorescent protein (iRFP). *In vitro* and *in vivo* characterization demonstrated that CHIKV-iRFP retained similar replication and virulence phenotypes to its parental virus. Neonatal BABL/c mice and IFNAR^−/−^ A129 mice were highly susceptible to CHIKV-iRFP infection. Following intracranial (i.c.) inoculation, CHIKV-iRFP efficiently replicated and disseminated into whole body, resulting in rapid death in an age-dependent manner. Remarkably, upon footpad injection, CHIKV-iRFP readily disseminated from footpad to head and whole skeleton, with a specific tropism for bone marrow. Taken together, this novel reporter virus provides a powerful tool to track real time CHIKV replication and to test the *in vivo* efficacy of vaccines and antiviral therapeutics.

## Introduction

Chikungunya virus (CHIKV), which belongs to the genus *Alphavirus* of the family *Togaviridae*, is an important human pathogen transmitted by *Aedes aegypti* and *Ae. albopictus* mosquitoes [1]. It was first isolated from the blood of an infected patient in Tanzania in 1953 [2]. Before 2004, CHIKV was considered nonfatal, and the common clinical symptoms are fever, rash, headache, arthralgia and myalgia. However, increasing number of severe or fatal cases associated with CHIKV infection have been reported since 2004, indicating that this virus may have become more virulent [3]. CHIKV is a small, enveloped virus with a message-sense RNA genome that encodes four non-structural proteins (nsP1-nsP2-nsP3-nsP4) and five structural proteins (C-E3-E2-6K-E1) [4]. A substitution of alanine to valine at amino acid position 226 (A226 V) in the E1 envelope glycoprotein was identified in viral isolates obtained during the major outbreaks in 2005–2007 [5]. It was demonstrated that this mutation significantly increased its fitness for *Ae. Albopictus* mosquitoes and most likely contributed to the epidemic of CHIKV [6]. CHIKV is now considered a re-emerging pathogen as numerous outbreaks have been reported in different non-endemic areas [7]. Currently, there is no available antiviral therapeutics against CHIKV infection.

To study viral infection in animal model, luciferase has been exploited to construct reporter viruses for *in vivo* imaging. This method has been applied for different viruses such as dengue virus [8], Japanese encephalitis virus [9], influenza virus [10], Sindbis virus [11], Sendai virus [12], herpes simplex virus 1 (HSV-1) [13] and vaccinia virus [14]. Luciferases from different species catalyse the oxidation of various substrates, producing bioluminescence in live cells or animals. Due to the lack of endogenous bioluminescent reactions in mammalian tissue, luciferase imaging offers a relatively low background tissue signal [11]. Compared with the luciferase imaging, the prominent advantage of fluorescent proteins (FPs) imaging is that it does not require injection of exogenous substrates for imaging [15]. However, the use of the conventional FPs derived from the green fluorescent protein family (GFP-like FPs) for deep tissue visualization is limited due to the spectral overlap of GFP with tissue autofluorescence [16]. Recently, the discovery of a phytochrome-based near infrared fluorescent protein (iRFP) has paved the way for the utilization of FPs in *in vivo* imaging. In the optical window of iRFP (from 650 to 900 nm), mammalian tissues are relatively transparent. Thus, iRFP-based FPs imaging can overcome the limitations of imaging with conventional GFP-like FPs, producing substantially higher signal-to-background ratio in animal models and allows *in vivo* deep-tissue imaging. In addition, iRFP is stable and noncytotoxic *in vivo*, making it a promising variant for organism labelling [16–18]. However, it is still an almost untouched field in terms of the application of iRFP in either *in vitro* or *in vivo* virus assays except for one case reported recently in rabies virus [19].

Here, we report a novel CHIKV-iRFP reporter virus for *in vivo* imaging in real-time manner without the addition of exogenous substrate. Dose-dependent fluorescence intensities were observed with increasing amounts of virus inoculation in native mice. The viral replication dynamics were permitted to monitor in the same mouse throughout the course of infection. The dissemination of CHIKV-iRFP in the entire skeletal system was detected. The viral loads in different tissues correlated well with the intensity of iRFP fluorescence. CHIKV-iRFP reporter virus offers a powerful tool to study the pathogenicity of CHIKV and to evaluate the effectiveness of vaccines and the potential antiviral agents.

## Materials and methods

### Cell culture, viruses and mice

Baby hamster kidney (BHK-21) cells were grown in Dulbecco's modified Eagle's medium (DMEM) containing 10% foetal bovine serum (FBS), 100 U/ml penicillin and 100 µg/ml streptomycin of in 5% CO_2_ at 37°C. Wild-type (WT) CHIKV and CHIKV-iRFP reporter viruses were generated from their corresponding infectious cDNA clones. Mouse strains used in this study included suckling BALB/c mice and 3–4-week-old 129/Sv/Ev mice deficient in type I IFN receptors (A129 mice). All animal experiments were performed in strict accordance with the guidelines of the Chinese Regulations of Laboratory Animals (Ministry of Science and Technology of People's Republic of China) and Laboratory Animal-Requirements of Environment and Housing Facilities (National Laboratory Animal Standardization Technical Committee). The experimental protocols were approved by the Animal Experiment Committee of Beijing Institute of Microbiology and Epidemiology, Beijing, China.

### Plasmid construction

The infectious clone of pACYC-CHIKV plasmid [4] was used as the backbone to construct CHIKV-iRFP plasmid. The cDNA sequence of iRFP [17] was chemically synthesized by commercial (Sangon Biotech, Shanghai, China) and introduced into pACYC-CHIKV by fusion PCR. Briefly, the iRFP reporter gene was fused to the site between nsP4 and capsid (C) genes in the full length CHIKV genome using the following primer pair: Forward, 5′-TTG GGC GCG CCA TGG CGG AAG GAT CCG TCG CC-3′; Reverse, 5′-TCC TTA ATT AAC TAC TCT TCC ATC ACG CCG ATC-3′, and another subgenomic (SG) promoter was introduced immediately downstream of iRFP gene in favour of recombinant virus replication. All the constructs were verified by DNA sequencing.

### RNA transcription and transfection

The plasmids of WT CHIKV and CHIKV-iRFP reporter viruses were linearized by *Bam*HI followed by *in vitro* transcription using a T7 mMESSAGE mMACHINE kit (Ambion) according to the manufacturer's protocols. The transcribed RNAs were electroporated into 8 × 10^6^ BHK-21 cells as described previously [4]. After transfection, the supernatants were collected at different time points and aliquoted at −80°C.

### Immunofluorescence assay (IFA)

BHK-21 cells transfected with WT CHIKV or CHIKV-iRFP RNA were seeded on coverslips. The transfected cells were collected at different time points post-transfection and fixed with cold 5% acetone in methanol for 10 min at room temperature. The fixed cells were then incubated with the rabbit polyclonal antibody against CHIKV E2 protein for 1 h at room temperature. After washing three times with PBS, the cells were incubated with goat anti-rabbit IgG conjugated with FITC. The coverslips were mounted on glass slides with 90% glycerol after three washes with PBS. Images were captured under a fluorescent microscopy (Nikon Eclipse TE2000). Bone marrow cell was extracted from imaged skeletons [20,21] and smeared on slides. Bone marrow was fixed by 5% cold acetic acid in methanol for 10 min at room temperature. The fixed bone marrow was washed three times with PBS and then incubated with mouse polyclonal anti-CHIKV antibody (1:200 dilution with PBS) for 1 h. After washing with PBS for three times, the bone marrow was incubated with a secondary antibody (goat anti-mouse IgG H&L-Alexa fluor 488 preabsorbed, Abcam, Cat # ab150117; 1:200) at room temperature for 1 h. After three times of washing with PBS, the slide was examined by a fluorescent microscope.

### Plaque assays

BHK-21 cells (2 × 10^5^ cells per well in a 12-well plate) were infected with 10-fold serial dilutions of WT or CHIKV-iRFP viruses for 1 h at 37°C and then overlaid with DMEM containing 2% FBS and 2% methyl cellulose. The cells were fixed with 3.7% paraformaldehyde (PFA) and stained with 1% crystal violet at 3 days post infection. The plaque numbers and morphology were reordered after washing with tap water.

### Viral growth kinetics

BHK-21 cells (3 × 10^5^ cells per well in a 6-well plate) were infected at a multiplicity of infection (MOI) of 0.01. The cell culture medium was then collected at the indicated time points post infection and was subjected to plaque assay to determine viral titres.

### Animal experiments

For correlation studies, one-day-old BALB/c mice were inoculated intracranially (i.c.) with 0.01, 0.05, 0.1, 0.5, 1, 5 (×10^3^ PFU) of CHIKV-iRFP diluted in 20 µl DMEM. The infected animals were imaged at 24 hpi, and the fluorescence signals were quantified and plotted against the amounts of inoculated viruses.

For the age-dependent virulence tests, three groups of BALB/c mice from three dams (*n* = 5 or 6), ranging from one-day-old to seven-day-old, were infected through i.c. injection with CHIKV-iRFP (10^4^ PFU) diluted in 20 µl DMEM. Infected animals were monitored for morbidity and mortality and weighted every day and imaged at the indicated time points.

For the survival study, groups of four-week-old A129 mice (*n* = 8) were infected through i.c. injection with CHIKV-iRFP or CHIKV (10^3^ PFU) diluted in 20 µl DMEM and were monitored daily for 7 days to assess morbidity and mortality. Moribund mice (*n* = 2) infected with CHIKV-iRFP or CHIKV, along with mock-infected A129 mice, were euthanized at 5 dpi and tissues were harvested. The isolated tissues were then weighed, ground in DMEM and stored at −80°C for viral burden detection. Muscles were completely removed from bones [22] and isolated skeletons were then imaged.

### Histology

For histology assay, the collected tissues including liver, spleen, brain and paw were fixed in 4% PFA (pH 7.4) and embedded in paraffin. After stained with haematoxylin and eosin (H&E), the sections were viewed by light microscopy.

### *In vivo* imaging

All the imaging was performed using IVIS Spectrum instrument (Perkinelmer) in epifluorescence mode equipped with 675/30 nm excitation and 720/20 nm emission filters. Head fur of A129 mice was removed using a depilatory cream, and the mice were under anesthesia throughout the imaging procedure. For *ex vivo* tissues imaging, isolated tissues from CHIKV-iRFP or mock infected mice were imaged after dissection. Fluorescence signals in regions of interest (ROIs) were quantified using Living Image 3.0.

### Statistical analysis

The significance of differences in survival rates was determined by Kaplan-Meier analysis using Prism version 4.00 for Windows (GraphPad). All titration data were log_10_ transformed and compared using unpaired Student's *t*-test. In determining the correlation between PFU and fluorescence values, curves were analysed using Pearson correlation with 95% confidence interval.

## Results

### Construction and characterization of CHIKV-iRFP reporter virus

**Figure 1.**
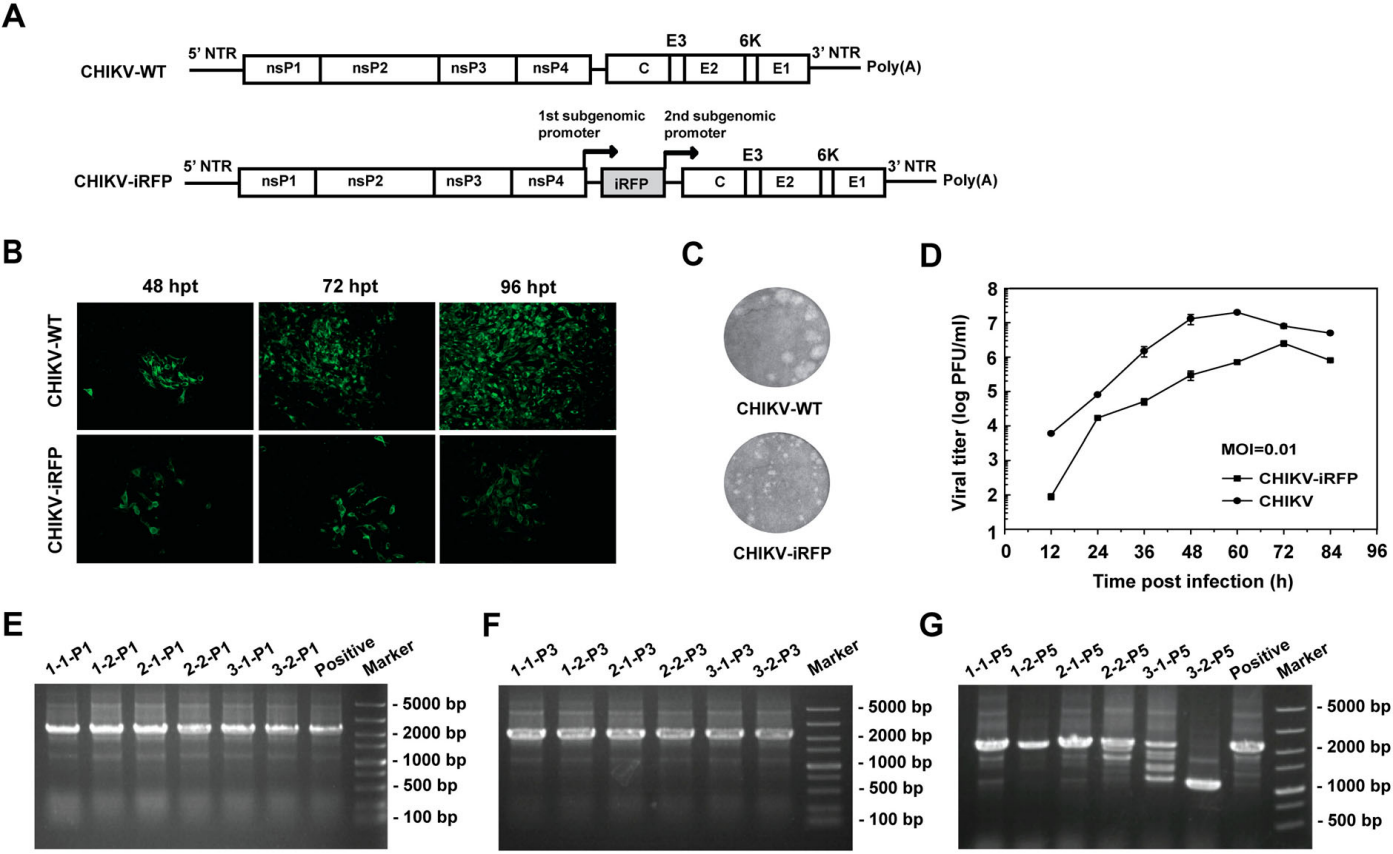
Construction and characterization of CHIKV-iRFP. (A) Schematic representation of WT CHIKV and CHIKV-iRFP reporter viruses. An infectious cDNA clone of CHIKV was used as a backbone for the construction of CHIKV reporter virus. The expression of iRFP reporter gene was driven by a duplicate subgenomic (SG) promoter. Arrows represent SG promoters. (B) IFA analysis of viral E2 expression in BHK-21 cells transfected with *in vitro* transcribed genome-length RNAs of WT CHIKV and CHIKV-iRFP. (C) Plaque morphology of WT CHIKV and CHIKV-iRFP reporter viruses in BHK-21 cells. (D) Comparison of the growth kinetics of WT CHIKV and CHIKV-iRFP reporter viruses. The growth of WT CHIKV and CHIKV-iRFP were compared at an MOI of 0.01 in BHK-21 cells. Three independent experiments were performed in duplicate, and the representative data were presented. Error bars represent standard deviations. (E–G) Detection of the iRFP reporter gene during viral passage. Viral RNAs were extracted from the cells of P0 to P5 passage, respectively. RT-PCR was performed with a primer pair locating between the region of nsP4 and C. The numbers of time points-samples-passage were denoted on the top of each lane.

The CHIKV infectious clone pACYC-CHIKV was used as a backbone to construct CHIKV reporter virus (CHIKV-iRFP) based on a dual subgenomic (sg) promoter method. Briefly, Additional sg promoter was used for iRFP cassette expression that was inserted before structural genes as depicted in Figure 1(A). A T7 promoter was also engineered at the 5′-end of viral genome sequence for *in vitro* transcription.

Equal amount of WT CHIKV and CHIKV-iRFP genomic RNAs transcribed from the infectious cDNA clones were transfected into BHK-21 cells. The abilities of viral replication were compared by IFA using anti-E2 rabbit polyclonal antibody (Figure 1(B)). Increasing amount of IFA positive cells were observed in both RNAs-transfected cells although CHIKV-iRFP produced less IFA positive cells than WT CHIKV at each time point post-transfection (Figure 1(B)). Additionally, plaque morphologies and viral growth curves were compared between WT CHIKV and CHIKV-iRFP. As shown in Figure 1(C), CHIKV-iRFP produced smaller plaques than WT CHIKV. Consistent with IFA results, CHIKV-iRFP could replicate efficiently although the viral titres were around 10-fold lower than those of WT CHIKV as quantified by plaque assay at each time point (Figure 1(D)). In general, the data showed that the CHIKV-iRFP is replication-competent and infectious, although the viral replication efficiency is slightly lower than that of WT CHIKV.

After demonstrating replication-competency of reporter viruses, the stability of CHIKV-iRFP was then tested in BHK-21 cells through blind passage for five rounds and the multiplicity of infection (MOI) used at each passage was 0.05. The culture supernatants collected from CHIKV-iRFP RNA transfected BHK-21 cells were defined as P0, and the cell cultures derived from each passage were defined as P1 to P5, respectively. The P0 CHIKV-iRFP culture supernatants obtained at 3 different time points were subjected to blind passage in duplicate. The viral RNAs derived from each passage were used to perform RT-PCRs with the primer pair of CHIKV-7376-F and CHIKV-8498-R to amplify the fragment between nsP4 and capsid that covers the region of the inserted reporter gene. 1.1 and 2.2 Kb RT–PCR products should be detected for WT and reporter viruses, respectively. As shown in Figure 1(E–G), CHIKV-iRFP reporter virus was considerably stable for at least 3 serial passages since 2.2 Kb RT–PCR products were detected at P3 passage in all three independently passaged viruses (Figure 1(F)). The iRFP reporter gene began to lose from P5 passage as indicated by multiple < 2.2 Kb RT–PCR products (Figure 1(G)). Overall, such phenotypes of CHIKV-iRFP reporter virus indicate that this reporter virus can be used to study the dynamics of CHIKV infection.

### Correlation between fluorescence intensity and viral replication capability of CHIKV-iRFP

**Figure 2.**
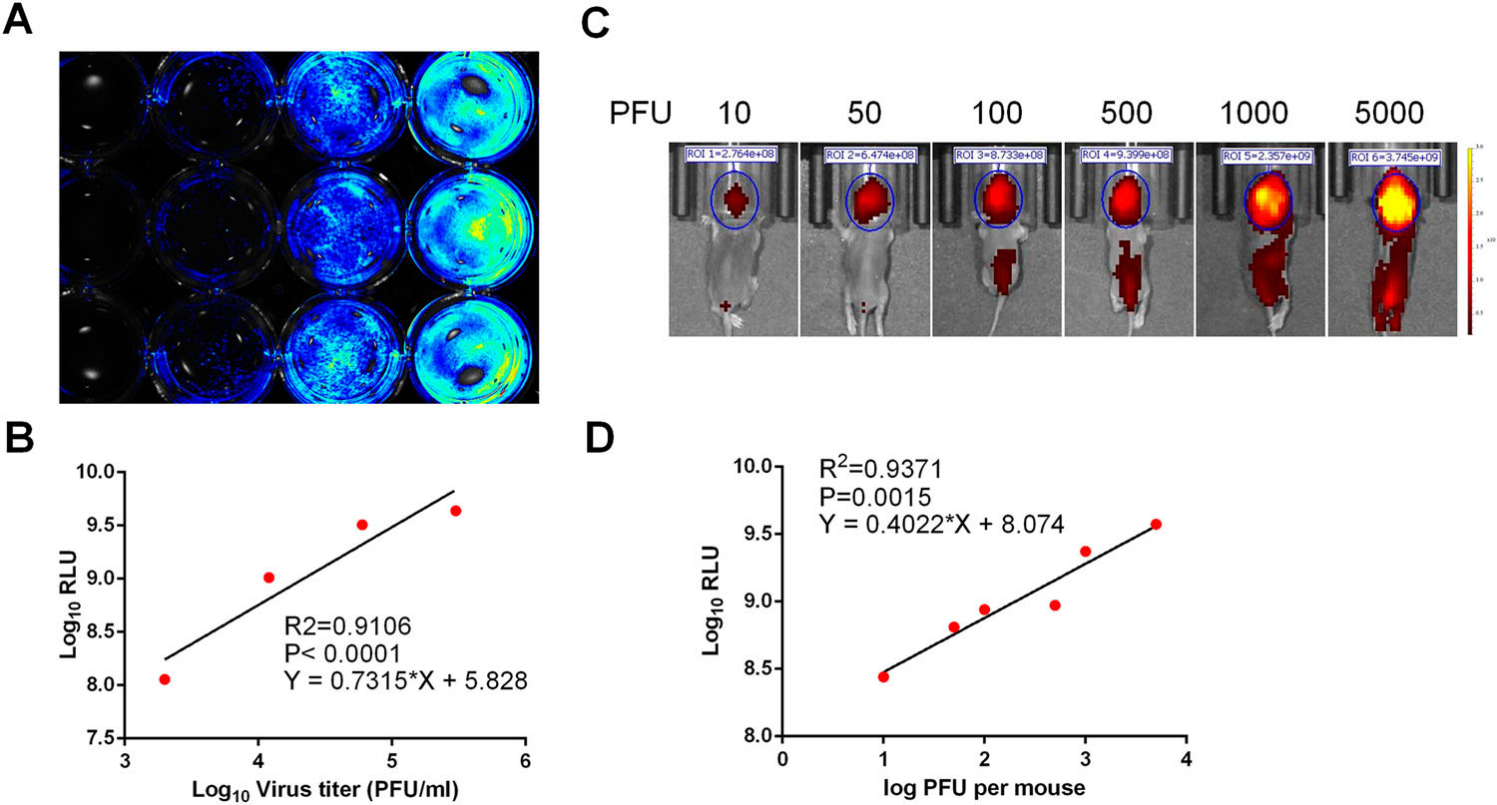
Correlation between fluorescence intensity and viral replication capability of CHIKV-iRFP. (A,B) *In vitro* correlation between fluorescence intensity and viral replication capability. BHK-21 cells in a 12-well plate were infected with CHIKV-iRFP at the 0.1, 0.5, 2.5 MOI, respectively. At 48 hpi, the plate was imaged (A), and the culture media were collected and subjected to plaque assay. Fluorescence radiant efficiency quantified by Living Image software was plotted against viral titre in the culture media (B). (C,D) *In vivo* correlation between fluorescence intensity and viral replication capability. One-day-old BALB/c mice were infected through i.c. injection with the indicated doses of CHIKV-iRFP. Representative images from three mice were shown at 24 hpi using IVIS Spectrum instrument equipped with 675/30 nm excitation and 720/20 nm emission filters. The colour bar indicates the fluorescence radiant efficiency (C). Total fluorescence radiant efficiency of the head areas of the iRFP expressing mice was plotted against inoculation dose (D).

To determine whether the expression of iRFP could be used as readout of viral replication, naïve BHK-21 cells in a 12-well plate were infected with CHIKV-iRFP at different MOIs. At 48 h post infection (hpi), the plate was used to quantify the fluorescent intensities with IVIS machine. A dose-dependent increase in iRFP signals was observed in BHK-21 cells infected with increasing amounts of CHIKV-iRFP (MOI of 0.1, 0.5 and 2.5) and no fluorescence signal was detected in naïve BHK-21 cells (Figure 2(A)). A good correlation between the amount of viral inoculum and fluorescence signal value was observed (*R*^2 ^= 0.9106, Figure 2(B)). These data suggested that iRFP expression could be used to monitor viral replication and quantify viral load of CHIKV-iRFP in cells culture.

To examine the feasibility of CHIKV-iRFP for *in vivo* imaging, one-day-old neonatal BALB/c mice were i.c. injected with different doses of CHIKV-iRFP and subjected to iRFP imaging at 24 hpi. As shown in Figure 2(C), fluorescence signals were observed within the brain in a dose-dependent manner. Especially, in animals inoculated with CHIKV-iRFP over 100 PFU, fluorescence signals expanded from the brain to the spinal cord in a dose-dependent manner. We further examined the correlation between iRFP signals and viral titres in the CHIKV-iRFP infected mice. Linear regression analysis showed a good correlation (*R*^2 ^= 0.9371) between the amount of viral inoculum and the intensity of fluorescence signal (Figure 2(D)). These results indicated that the iRFP imaging in mice can accurately reflect the status of viral infection in real time.

### Visualization of CHIKV-iRFP infection in suckling mice

**Figure 3.**
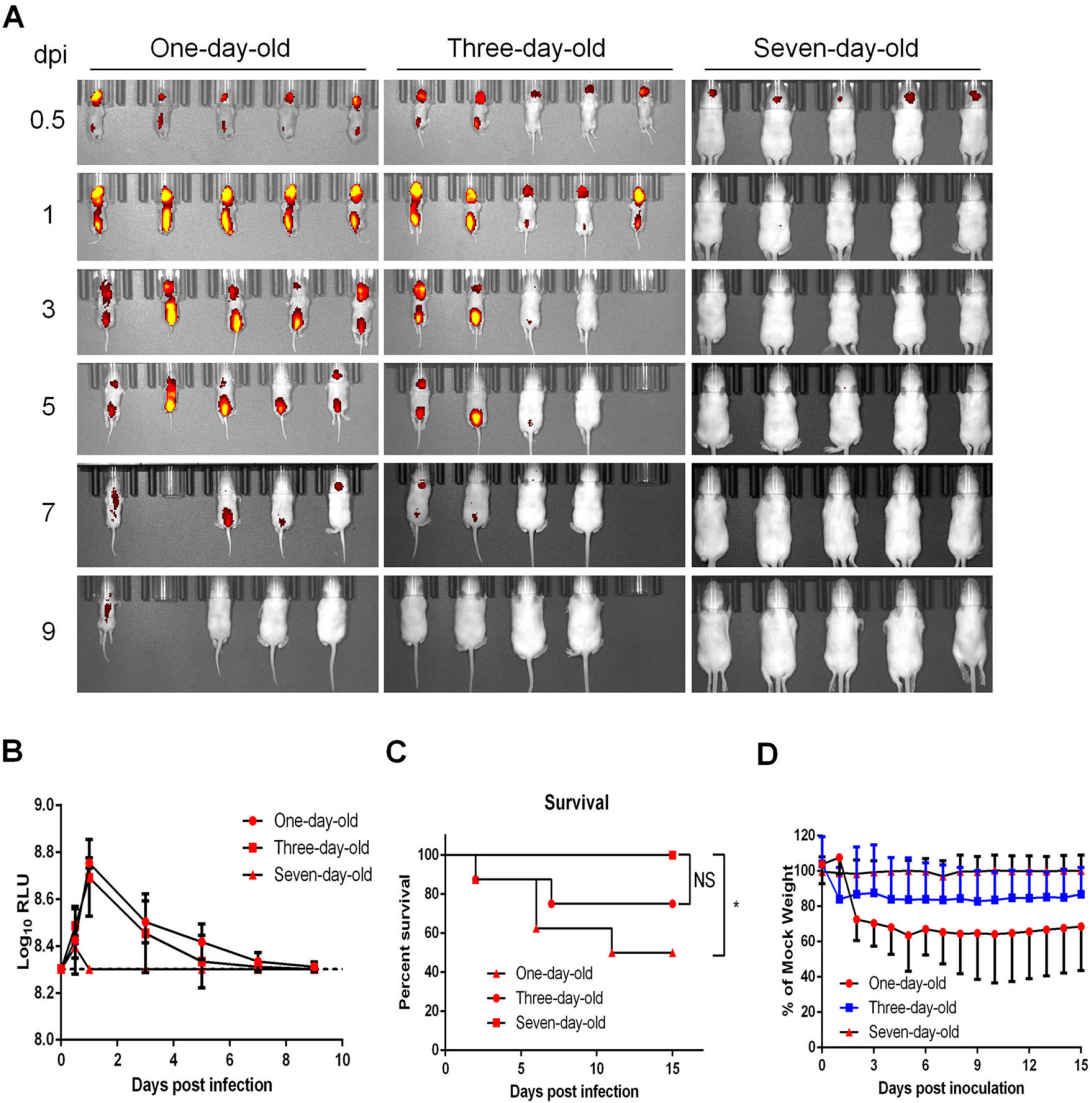
Age-dependent replication in suckling BALB/c mice. (A) Time-course whole-body fluorescence images in suckling BALB/c mice at different ages. Suckling BALB/c mice (*n* = 5 or 6) of indicated ages were infected through i.c. injection with CHIKV-iRFP (10^4^ PFU) diluted in 20 μl of DMEM and were imaged at the indicated time points. (B) Total fluorescence radiant efficiency of the head areas of the iRFP expressing mice in (A) was quantified using Living Image software. (C) Survival curve of the iRFP expressing mice in (A). (D) Time-course body weight changes in suckling mice in (A).

CHIKV is a typical neurotropic arbovirus with significant virulence in suckling mice. Here, to visualize the *in vivo* replication kinetics of CHIKV-iRFP, BALB/c suckling mice at different ages (1-, 3-, and 7-day-old) were i.c. inoculated with 10^4^ PFU of CHIKV-iRFP followed by iRFP imaging. In one-day-old and three-day-old mice infected with CHIKV-iRFP, strong fluorescence signals were observed at the inoculated site and spinal line as early as 0.5 days post infection (dpi), peaked at 1 dpi, decayed till 7 dpi, and finally vanished at 9 dpi. In contrast, only weak fluorescence was detected at the local site of inoculation in seven-day-old mice at 0.5 dpi, and afterwards, declined rapidly to basal levels (Figure 3(A,B)), suggesting that 7-day-old mouse has become resistant to CHIKV-iRFP. Consistently, the neurovirulence phenotypes in different groups of mice perfectly matched the fluorescence signals. Half of one-day-old mice, and 25% of the 3-day-old mice, died with typical neurological symptoms infection within 15 days, whereas all seven-day-old mice survived without any clinical symptoms following CHIKV-iRFP infection (Figure 3(C)). The body weight of one-day-old and three-day-old mice decreased upon CHIKV-iRFP inoculation compare with seven-day-old mice (Figure 3(D)). These results illustrated the infection and spread of CHIKV-iRFP following i.c. infection in suckling mice at different ages.

### Visualization of CHIKV-iRFP infection and dissemination in a129 mice

**Figure 4.**
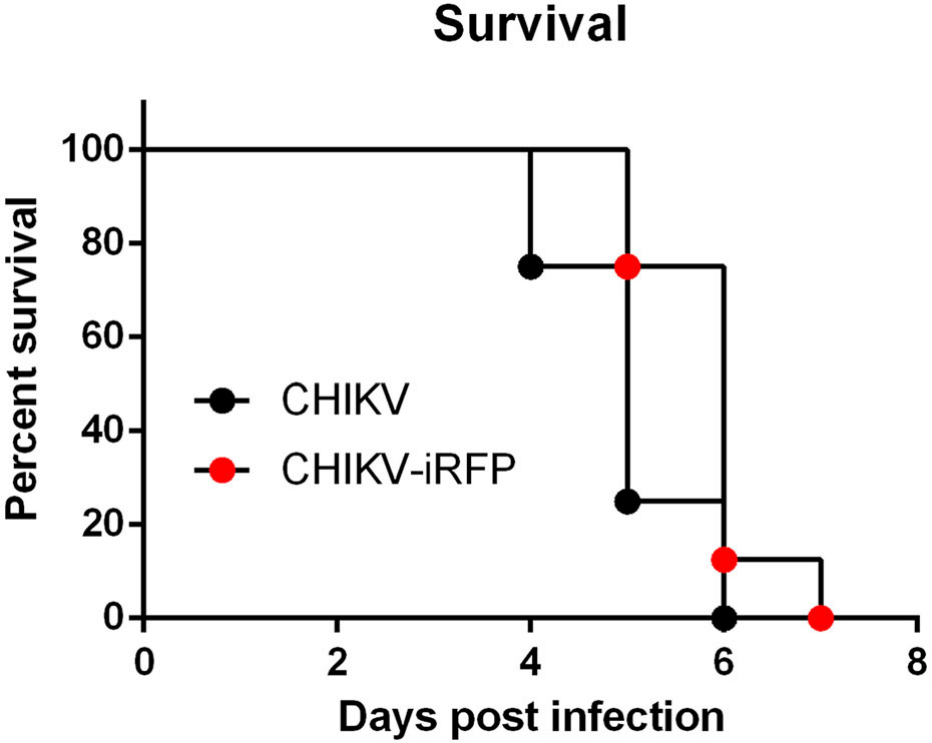
Virulence of CHIKV-iRFP and the parental CHIKV. Four-week-old A129 mice (*n* = 8) were infected through i.c. injection with CHIKV-iRFP or CHIKV (10^3^ PFU) diluted in 20 μl of DMEM and were monitored daily for 7 days to assess morbidity and mortality. Log-rank (Mantel-Cox) test was performed (*p < 0.05).

A129 mice have been well documented as a useful animal model for studying CHIKV pathogenesis [23,24]. Thus, we further characterized the *in vivo* replication and dissemination of CHIKV-iRFP in A129 mice. We first compared the mortality of CHIKV-iRFP and its parental CHIKV in A129 mice. To this end, four-week-old A129 mice were infected through i.c. injection with CHIKV-iRFP or CHIKV (10^3^ PFU) and monitored disease status daily for 7 days. As shown in Figure 4, both CHIKV-iRFP and CHIKV killed all A129 mice within 7 days.

**Figure 5.**
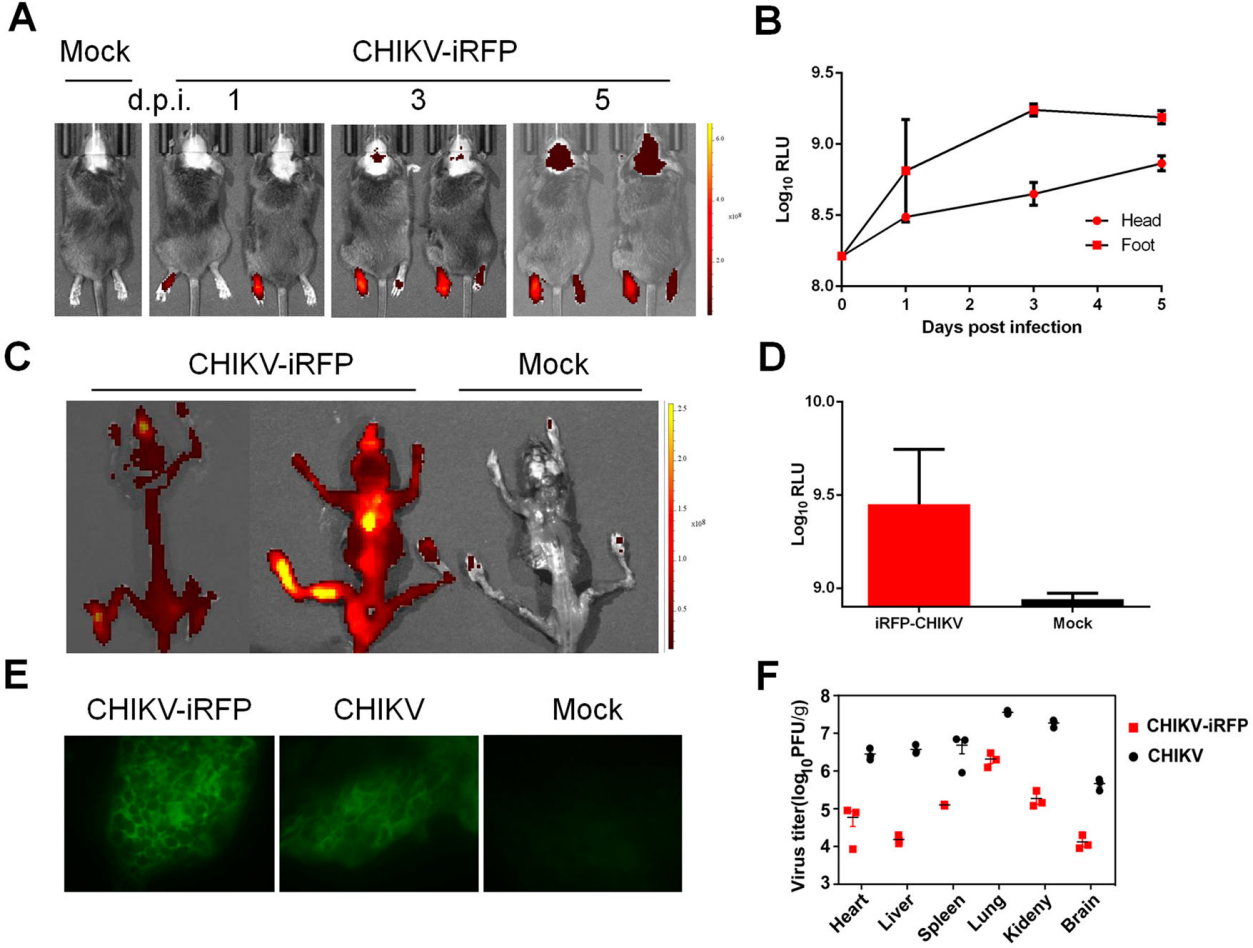
*In vivo* imaging of CHIKV-iRFP dynamics and localization. (A) Viral dynamics and localization in infected A129 mice. A129 mice were infected through left footpad with CHIKV-iRFP (10^3^ PFU) diluted in 50 μl of DMEM and imaged at the indicated time points with head fur removed using a depilatory cream. (B) Fluorescence signals of mice in (A) were quantified using Living Image software. (C) The skeletons were imaged after euthanasia of dying mice. (D) Fluorescence signals of mice in (C) were quantified using Living Image software. (E) Bone marrow cells were harvested from CHIKV-iRFP, CHIKV and mock infected A129 mice after euthanasia and IFA was performed on the bone marrow smears using anti-CHIKV polyclonal antibody. (F) A129 mice were euthanized at 5 dpi and tissues were isolated and homogenized. Plaque assays were performed to quantify viral load in tissues.

Furthermore, iRFP imaging showed that following the injection at the left footpad of A129 mice, CHIKV-iRFP disseminated to the non-injected (right) footpad and to the head at the 3 dpi, and the intensity of fluorescence signals reached up to approximately 9×Log_10_RLU at 5 dpi (Figure 5(A,B)). We then further examined the viral distribution in whole skeleton. Strikingly, strong fluorescence signals were seen in all CHIKV-iRFP infected animals (Figure 5(C,D)). Furthermore, to make sure the site of CHIKV-iRFP replication, primary bone marrow cells were isolated and subjected to IFA. The results indicated that both bone marrow smears harvested from WT and CHIKV-iRFP-infected mice were positive for viral antigen (Figure 5(E)), in agreement with previous findings that CHIKV mainly targets the bone and joint-associated organs [25]. The viral load in the tissues from A129 mice after WT and CHIKV-iRFP infection was also measured via plaque assays. The results showed that the viral burden in tissues from mice inoculated with CHIKV-iRFP was lower than that of mice inoculated with the WT CHIKV. A considerable amount of viral load was detected in these tissues, including heart, liver, spleen, lung, kidney and brain. Taken together, these data indicate that CHIKV-iRFP reporter virus allows us to visualize viral replication and spread in infected A129 mice, representing a powerful tool for pathogenesis study and developing the antiviral therapeutics.

**Figure 6.**
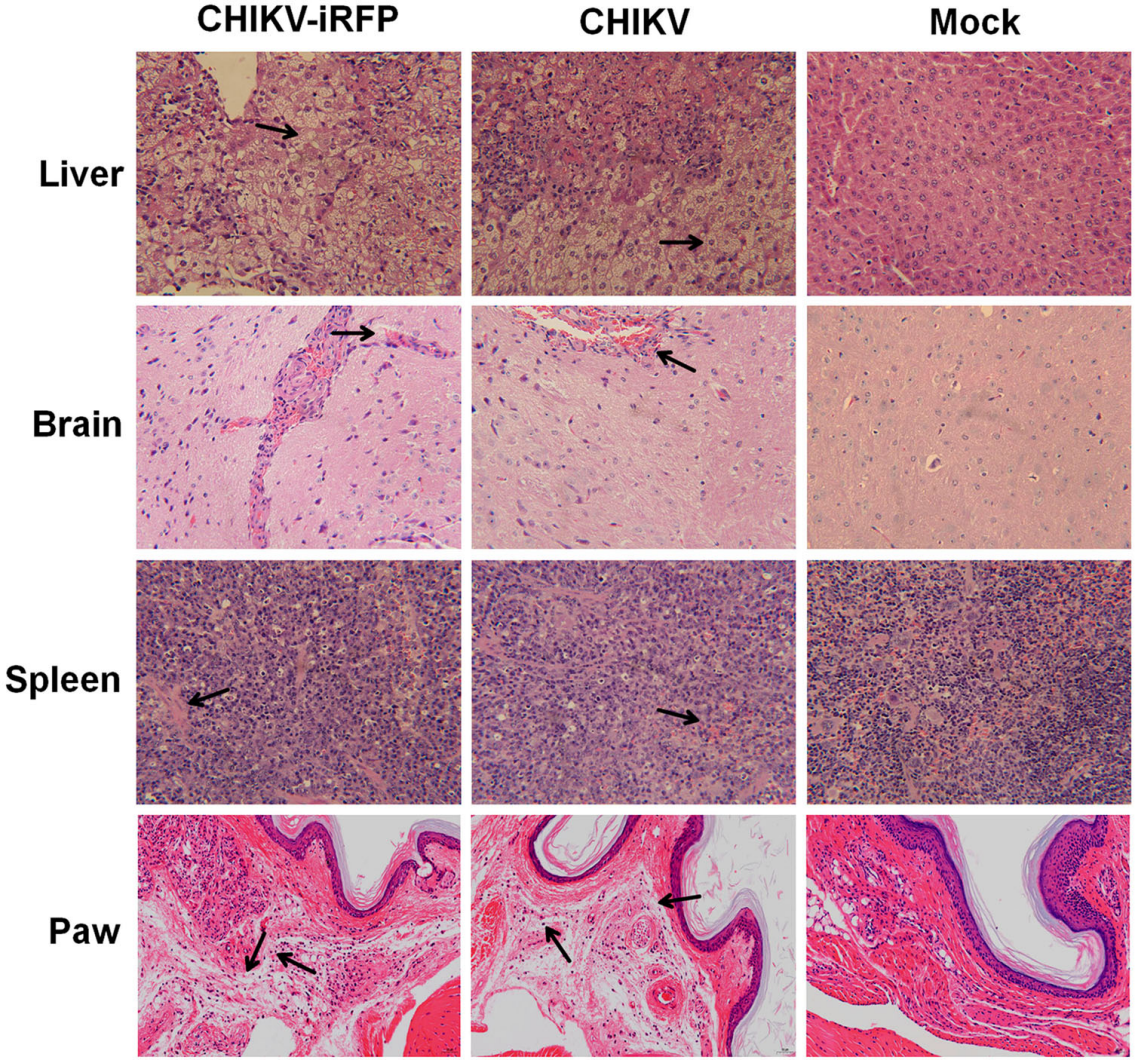
Histology of tissue sections from mice infected with CHIKV and CHIKV-iRFP. The tissues (liver, brain, spleen and paw) from the mice infected with CHIKV-iRFP, WT CHIKV or PBS were collected at 5 dpi and then stained via the haematoxylin and eosin method (H&E) 400X. The arrows indicated the typical histological lesions in the detected tissues.

To further compare histopathological lesions induced by WT and CHIKV-iRFP viruses in A129 mice, the tissues of liver, brain, spleen and paw were collected for histopathological analysis. As shown in Figure 6, these tissues showed similar patterns of pathology in WT and CHIKV-iRFP-infected mice compared with the mock-infected mice. There were severe liver cell oedema and focal cell necrosis in the liver isolated from infected mice. The spleens from infected mice exhibited extensive haemorrhage and congestion. In addition, mild lesions with lymphoid nodule occasionally were observed in the brain of both viruses-infected mice. The paws from both viruses-infected mice exhibited dermal oedema, connective tissue loose, with inflammatory cells scattered infiltration. Taken together, these results demonstrated that the pathological changes seen in the tissues were relatively consistent in CHIKV-iRFP-infected mice compared with the WT CHIKV-infected mice.

## Discussion

The re-merging of CHIKV with increased virulence requires a reliable method to quickly assess viral pathogenicity and evaluate the efficacy of antiviral therapeutics. In recent years, the development of reverse genetics for alphavirus has boosted the mechanistic studies of alphavirus replication and pathogenesis [1,26]. A number of CHIKV reporter viruses expressing different reporter genes have been developed to study viral replication and pathogenesis [25,27,28]. In this study, we developed a replication-competent CHIKV reporter virus (CHIKV-iRFP) encoding iRFP gene for noninvasive *in vivo* imaging.

The studies conducted in animal models are important for investigating *in vivo* viral replication, the pathogenesis of viral infection, and the efficacy of antiviral interventions [11]. Conventionally, this requires a large number of animals to acquire the data at different time points, and thus entails a lot of tedious work such as sacrificing animals, isolating organs and detecting viral titres. *In vivo* imaging based on reporter viruses, as an alternative approach, allows real-time tracking and quantifying viral replication and dissemination in the same animal without sacrificing animals. This technology not only greatly increases efficiencies of *in vivo* studies, but also can better show the temporal and spatial variability of the infection and improves data accuracy. Currently, it has been widely used in biological research with different purposes [29–31]. Palha *et al.* set up a novel CHIKV infection model in zebrafish using CHIKV-GFP reporter virus, where the virus dissemination in the whole body was live-imaged. In addition, it was found that the CHIKV infection also triggered a strong type-I interferon (IFN) response in zebrafish as in mammals [30]. An *in vivo* imaging of CHIKV infection model in mosquito was also performed using CHIKV-GFP reporter virus by enema delivery, showing an advantage than intrathoracic inoculation [31].

In comparison with WT virus, CHIKV-iRFP reporter virus displayed a slower growth rate and smaller plaque size (Figure 1(B,C)), indicating some levels of attenuation in cell culture. Consistently, data from *in vivo* virulence assay with A129 mice showed that the mortality rate of mice inoculated with CHIKV-iRFP was slightly lower than that of mice inoculated with the parental CHIKV (Figure 4). Indeed, it is a common event for many reporter viruses that the addition of reporter gene causes *in vitro* and *in vivo* attenuation of viral replication, such as DENV [32], JEV [9] and influenza A virus [10]. Nevertheless, CHIKV-iRFP produced more than 10^6^ peak viral titres at 72 hpi in cell culture and intensive fluorescence signals in either neonatal BABL/c or immunocompromised A129 mice. It allows us to further characterizing dynamic replication and dissemination of CHIKV in living mice.

Based on the imaging efficiency of CHIKV-iRFP reporter virus in mouse, it was shown that two factors, including age and functionality of type-I IFN signalling, are the key determinant for *in vivo* replication efficiency of CHIKV. This finding is consistent to previous studies [33,34] as well as the clinical data [34] in which the neonates and adults with immune defect are highly susceptible to CHIKV infection. Although we have not yet figured out the reason why the neonate status and a defect in type-I IFN favour *in vivo* viral replication, our study provides straightforward evidence that the CHKV infection severity is age- and immune-dependent. In addition, intensive fluorescence signals of CHIKV-iRFP in the muscles and bones of infected A129 mice were detected, further demonstrating CHIKV muscular and arthralgic tropism.

The insertion site of reporter gene in alphavirus genome is critical for the stability of reporter virus and the virulence in animals [27]. A common strategy used to construct alphavirus harbouring a reporter gene is to express the reporter gene via an additional subgenomic promoter located either immediate upstream of the authentic subgenomic promoter (5′-26S) or downstream of E1 protein (3′-DP) [27,28,35]. In comparison with 3′-DP, 5′-26S approach is considered to be more stable and minimize virulence loss [27,35]. We therefore utilized the 5′-26S strategy to construct CHIKV-iRFP vector in the current study (Figure 1(A)). This reporter virus appeared to be stable at least within three rounds of passage (Figure 1(E–G)).

In summary, it was shown that this novel CHIKV-iRFP reporter virus could be used for *in vivo* imaging in living small animals. Through investigating the imaging efficiencies of reporter virus in mice at different ages or with different immunity status, it was demonstrated that age and immunity levels of mice are the key factors for efficient *in vivo* replication of CHIKV. This well-established CHIKV-iRFP reporter virus system will facilitate the study of viral replication, pathogenesis and the development of antiviral therapeutics.
